# Editorial: Heat stress and immune responses in livestock: current challenges and intervention strategies

**DOI:** 10.3389/fvets.2024.1366274

**Published:** 2024-01-24

**Authors:** Mohanned Naif Alhussien, Jamal Hussen, Giovanna De Matteis

**Affiliations:** ^1^Reproductive Biotechnology, TUM School of Life Sciences, Technical University of Munich, Freising, Germany; ^2^Department of Microbiology, College of Veterinary Medicine, King Faisal University, Al Hofuf, Saudi Arabia; ^3^Research Centre for Animal Production and Aquaculture, Council for Agricultural Research and Economics, CREA, Rome, Italy

**Keywords:** heat stress, livestock, immune response, disease susceptibility, mitigation strategies

Heat stress poses significant challenges to the sustainability of livestock, adversely affecting their overall health and productivity. Insufficient thermal adaptation resulting from heat stress compromises immune functions and increases susceptibility to diseases (1). Despite its impact on various immune functions, the underlying mechanisms governing immune dysfunctions in response to heat stress remain inadequately characterized. This knowledge gap exists because heat stress induces significant changes in metabolism and modulates the functions of the highly interconnected nervous, endocrine, and immune systems. Moreover, the impact and severity of heat stress on immune response and health status are influenced by animal-related factors, such as species, breed, productivity, age, physiological stage, metabolism rate, and thermoregulatory mechanisms (2, 3). Therefore, understanding the underlying mechanisms through which heat stress impairs essential immune system functions and predisposes animals to diseases is crucial for enhancing immune system resilience and minimizing health disorders in livestock. This Research Topic delves into the intricate relationship between heat stress and immune function in livestock, and contemplates intervention strategies aimed at alleviating the negative impacts of heat stress. Ultimately, the objective is to improve animal welfare and maintain optimal productivity in the face of thermal stress.

Several articles of this Research Topic focused on the impact of heat stress on dairy cattle. Scatà et al. investigated the impact of hyperthermia on leukocyte survival and phagocytosis in bovine and buffalo leukocytes. The study has revealed notable variations in the modulation of blood leukocyte viability and functions between bovines and buffaloes in response to different levels of hyperthermia highlighting that buffaloes exhibit relatively higher thermal-adaptation compared to bovine cells. These findings not only have deepened our understanding of how heat stress affects immune responses in these two crucial species but have also pinpointed the specific degrees and durations of hyperthermia exposure that compromise specific immune functions in each species.

There is an intricate interplay between genetic factors and the immune response to heat stress. The potential for genetic-based interventions to alleviate its adverse effects has garnered increased interest in recent years. Worku et al. conducted a comprehensive analysis of the genetic basis of heat tolerance in dairy cattle, highlighting the roles of candidate genes and genomic regions in conferring heat tolerance. Additionally, the article investigates the immune response to heat stress in dairy cattle and examines potential breeding strategies to mitigate its adverse effects. The findings underscore the importance of understanding the genetic and immune mechanisms underlying heat stress in dairy cattle, emphasizing the potential for breeding programs to relieve its impact on animal welfare and productivity. Cartwright et al. analyzed the effects of heat stress on dairy cattle and various selection strategies to enhance resilience to heat stress. The review explores different selection strategies for conferring thermotolerance, including reduced milk production, crossbreeding with thermotolerant breeds, and selecting based on physiological traits and enhanced immune response. The article emphasizes the potential benefits of implementing effective selection strategies to improve thermotolerance and mitigate its adverse effects on animal welfare and productivity.

In a systematic review article, Morgado et al. conducted a pioneering synthesis, amalgamating existing knowledge derived from experiments conducted in controlled conditions to evaluate the impact of management strategies in addressing escalating heat stress challenges in ruminants. This assessment involved comparing effects in both thermoneutral and heat stress (HS) experimental conditions (i.e., unconditional of being in HS) and in HS experimental conditions only (i.e., conditional of being in HS). The results highlight that, although management measures, encompassing both adaptation and mitigation strategies, can enhance resilience to heat stress, their effectiveness is constrained under conditions of extreme heat stress, which are becoming increasingly prevalent. Heat stress induces glucocorticoids secretions which suppress certain aspects of the immune response to prevent excessive inflammation, though prolonged exposure may lead to immunosuppression and increased susceptibility to infections. Giaretta et al. investigated the impact of temperature and humidity on salivary cortisol and dehydroepiandrosterone concentrations in growing bulls under stress induced by performance test procedures. The results indicated that temperature-humidity index (THI) affected the levels of these steroids, particularly during short exposure. The findings suggest that environmental conditions, as indicated by THI, can impact the stress response in growing bulls, highlighting the importance of considering temperature and humidity effects in stress-related research and management practices for livestock.

Heat stress diminishes the antioxidant capacity and immunity of poultry, exerting a significant impact on their overall welfare, health, and production. Yu et al. have devised an algorithm for detecting heat stress in poultry, utilizing the FPN-DenseNet-SOLO model. This algorithm enables accurate and swift identification of poultry under heat stress conditions, particularly in complex environments. Their findings demonstrated the feasibility and effectiveness of the improved SOLOv2 network in detecting heat stress and enhancing the precise breeding of poultry.

This Research Topic significantly advances our understanding of the impact of heat stress on immune competence and offers diverse strategies to bolster livestock resilience in the face of escalating climate change-induced heat stress. Further research should focus on micro-environmental management intervention techniques such as ventilation, sprinkling, shading, and the administration of feed supplements like vitamins, minerals, probiotics, and essential oils. Understanding the molecular and cellular mechanisms through which heat stress compromises essential immune functions is critical. Especially investigating the role of heat stress-induced changes in the epigenetic landscape of the immune system and in the composition of the microbiome in the modulation of the immune system are of primary importance. This knowledge can unveil novel molecules and pathways that enhance heat resilience and immunity while sustaining livestock production. A prospective approach involves genetically selecting animals adapted to heat stress, aiming to enhance immunity to infection or vaccination without compromising production. Future studies should also explore the impact of heat stress on pathogen persistence and survival, the geographical range and abundance of vectors, and the subsequent effects on immune responses in livestock. By delving into these unexplored realms, we can develop comprehensive strategies to fortify livestock against the challenges posed by climate change-induced heat stress.

## Author contributions

MA: Writing—original draft, Writing—review & editing. JH: Writing—review & editing. GD: Writing—review & editing.
